# Rational Design and Characterization of Symmetry-Breaking Organic Semiconductors in Polymer Solar Cells: A Theory Insight of the Asymmetric Advantage

**DOI:** 10.3390/ma14216723

**Published:** 2021-11-08

**Authors:** Zezhou Liang, Lihe Yan, Jinhai Si, Pingping Gong, Xiaoming Li, Deyu Liu, Jianfeng Li, Xun Hou

**Affiliations:** 1 Key Laboratory of Physical Electronics and Devices of the Ministry of Education & Shaanxi Key Lab of Photonic Technique for Information, School of Electronic Science and Engineering, Faculty of Electronic and Information Engineering, Xi’an Jiaotong University, Xi’an 710049, China; zezhouliang@foxmail.com (Z.L.); jinhaisi@mail.xjtu.edu.cn (J.S.); gpbxnl@163.com (P.G.); houxun@mail.xjtu.edu.cn (X.H.); 2 School of Materials Science and Engineering, Lanzhou Jiaotong University, Lanzhou 730070, China; ljfpyc@163.com; 3 School of Chemistry, Beihang University, Beijing 100191, China; lixiaoming617@hotmail.com; 4 Department of Materials Science and Engineering, Ocean University of China, Qingdao 266100, China; lunlunyu@126.com

**Keywords:** DFT, TD–DFT, asymmetric structure, structure–performance relationships, polymer solar cells

## Abstract

Asymmetric molecule strategy is considered an effective method to achieve high power conversion efficiency (PCE) of polymer solar cells (PSCs). In this paper, nine oligomers are designed by combining three new electron-deficient units (unit_A_)—n1, n2, and n3—and three electron-donating units (unit_D_)—D, E, and F—with their π-conjugation area extended. The relationships between symmetric/asymmetric molecule structure and the performance of the oligomers are investigated using the density functional theory (DFT) and time-dependent density functional theory (TD–DFT) calculations. The results indicate that asymmetry molecule PEn2 has the minimum dihedral angle in the angle between two planes of unit_D_ and unit_A_ among all the molecules, which exhibited the advantages of asymmetric structures in molecular stacking. The relationship of the values of ionization potentials (IP) and electron affinities (EA) along with the unit_D_/unit_A_ π-extend are revealed. The calculated reorganization energy results also demonstrate that the asymmetric molecules PDn2 and PEn2 could better charge the extraction of the PSCs than other molecules for their lower reorganization energy of 0.180 eV and 0.181 eV, respectively.

## 1. Introduction

Polymer solar cells (PSCs) have attracted wide attention for their low-cost, lightweight, flexible, and roll-to-roll fabricating characters [1,2,3,4,5,6,7], and could be one promising candidate for next generation renewable energy techniques [8,9]. Generally, a classical bulk-heterojunction (BHJ) PSC device is based on the active layer blended with the electron donor and electron acceptor materials [10,11,12]. The power conversion efficiency (PCE) of the PSCs has been further improved by many new organic semiconductors that have been well-designed and synthesized during the past decades [13,14,15,16,17,18]. For electron donor materials, the representative donor–acceptor (D–A) polymers, such as PM6 [19], PBDT-C [15], and D18 [20], have shown excellent performance. To date, the PCE is over 18% [20]. The D–A structure played a key role to improve device properties since their suitable bandgaps and energy levels can be easily tuned [21,22,23]. In addition, a strong intramolecular charge transfer (ICT) can form between donor and acceptor units, which leads to the expansion of the absorption spectrum and an increase in the absorption coefficient [24]. To obtain more advanced PSCs, many strategies were applied to design promising polymer donor materials, such as the introduction of halogen atoms, side-chain engineering, symmetry-breaking strategies, ternary copolymerization strategy, etc. [25,26].

Among these strategies, the symmetry-breaking design concept is attractive due to its excellent molecular packing and the low nonradiative energy loss of the asymmetric structure [27,28,29,30,31]. Many asymmetric D–A polymers have been investigated to gain insight into their advantages. Supramolecular locks could form among the blend films by introducing asymmetric halogen atoms and alkyl chains, which is beneficial for high fill fact (*FF*) [32]. Yang’s research group systematically inspected asymmetric benzodithiophene (BDT) units [31,33] and asymmetric backbones [30], which could efficiently modulate molecular aggregation and crystallinity. Based on asymmetric indenothiophene polymers designed and synthesized by Zheng et al., the PCE improved from 5.40% to 9.14% [34]. He et al. developed chlorination-atom-based asymmetric thiophene polymers [35], which could be fabricated as a thick film and a PCE of up to 9.11% was obtained. The concept of asymmetric structure is also widely applied in designing non-fullerene acceptors (NAFs). Bo et al. introduced asymmetric side chains to fused-ring acceptors. When blending with PBDB-T, the molecular packing could be enhanced effectively and the phase separation could be optimized [36]. Chen et al. designed new small molecule acceptors with asymmetric 4-alkyl-8-alkoxybenzo[1,2-b:4,5-b′]dithiophene as the central unit, and a high *FF* of 75% was achieved [37]. Sun et al. synthesized asymmetric ADA type NAFs by extending the conjugation of the indacenodithiophene backbone, which could significantly improve device performance [38]. Recently, Yan et al. developed an asymmetric end group strategy to tune ANFs energy levels, resulting in high PCE of over 17% [28]. The design concept of asymmetric structures has been widely applied and has experienced rapid progress.

**Figure 1:**
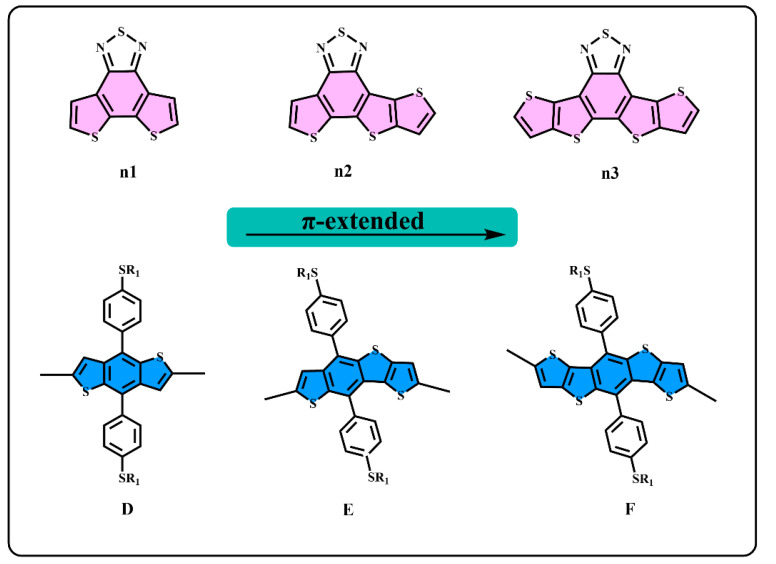
The structure of n1, n2, n3, D, E, and F.

**Figure 2:**
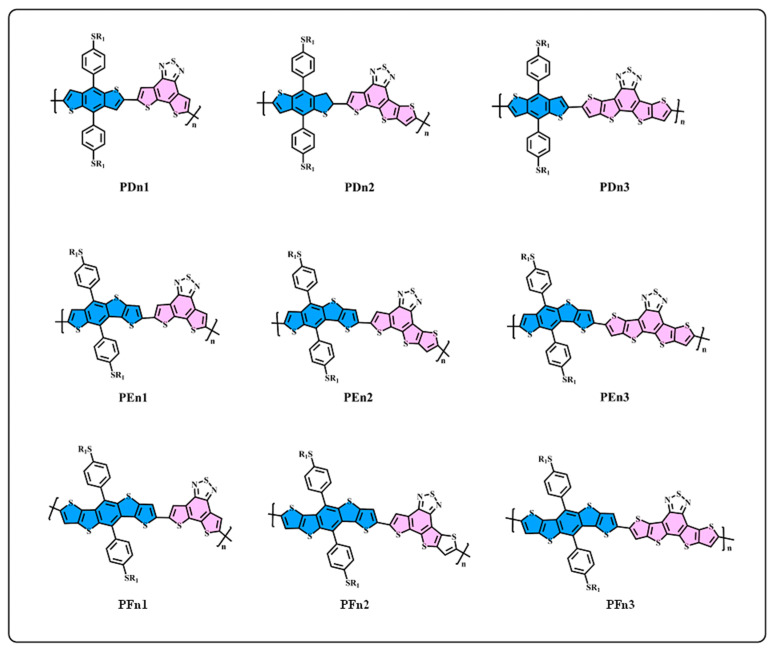
The structure of D–A oligomers: PDn1, PDn2, PDn3, PEn1, PEn2, PEn3, PFn1, PFn2, and PFn3.

However, the intrinsic nature of the superior properties of asymmetric structures have rarely been studied in-depth, which is very important for designing high-performance donor/acceptor materials. In this work, as one of the most basic schemes, the asymmetric structures on the π-extend of the conjugate plane of the donor/acceptor units are mainly investigated. We start from a general structure unit (n1), as displayed in Figure 1, and extend the molecular conjugate plane, then we obtained the n2 and n3 units, and the donor units D, E, and F using the same method. Based on the D–A alternative principle, nine polymers are obtained, as shown in Figure 2. The units from n1 to n3 and D to F are monomers with a π-conjugation area expanded gradually. n2 and E are asymmetric structure units, so that the nature of the asymmetric structure can be deeply investigated. Quantum chemical methods are an economical and effective way to understand and predict molecular structure and properties, which could avoid cumbersome synthesis [39,40]. The properties of energy level and of the optical and excited states are crucial for polymer donors for the photo-electric conversion processes [41]. In this paper, density functional theory (DFT) is employed to optimize the geometries and calculate energy levels, ionization potentials (IP), electron affinities (EA), and reorganization energies (λ) [42]. In addition, the time-dependent density functional theory (TD–DFT) is employed to calculate the absorption spectrum and excited states [43]. As a result, we found that the asymmetric molecule PEn2 shows the minimum dihedral angles that could be beneficial to form better molecular stacking. This shows the advantage of asymmetric structures. The properties of energy level, energy gap (*E*g), and the spectral absorption region could be regularly affected by changing the size of the conjugate region. The π-extend of the unit_D_/unit_A_ has a close relation with the IP/EA of the molecules and further influences the charge extraction of the PSCs device. We hope that this study can offer an important guideline to the design of asymmetric donor polymers.

## 2. Computational Methods

The molecular structure at the ground state and the molecular electrostatic potential (ESP) of all the monomers, as shown in Figure 1, were optimized in the gas phase under the B3LYP-(D3)BJ/6-311G(d) level [44,45,46]. The nine oligomers, as shown in Figure 2, were optimized in chloroform using the conductor-like polarizable continuum model (CPCM) [47], under the B3LYP-(D3)BJ/6-311G(d) level. The electronic absorption spectra and other excited-state properties of the oligomers were calculated using the time-dependent density functional theory (TD-DFT) under the CAM-B3LYP/6-311G(d) [42,48] level, employing chloroform (CPCM) as a solvent. All the alkyl side chains were replaced by methyl groups. All the calculations were performed by Gaussian 09 package [49]. Furthermore, the absorption spectra and electron transitions were analyzed by Multiwfn 3.7 [50,51].

## 3. Results and Discussion

### 3.1. Molecule Design

We generally design molecules with large conjugated polycyclic aromatic units for D–A alternative oligomers, which could effectively increase the coplanarity and π-electron delocalization, and could thus benefit for the light absorption and charge transportation [52]. As revealed in previous research [52,53], the unit_A_ blocks based on benzothiadiazole demonstrated excellent performance in organic electronics. In this work, we designed three benzothiadiazole-based moieties with the expansion of the conjugation area n1, n2, and n3, as shown in Figure 1. In addition, the phenyl substituted the benzo(1,2-b:4,5-b’)dithiophene (BDTP)-based two-dimensional (2D) electron-donating moieties have an advantage in the solubility, optical, and electrical properties [54]. Three electron-donating moieties based on BDTP (D, E, and F) were designed with gradual π-extend. n2 and E are asymmetric units. We further obtained PDn1, PDn2, PDn3, PEn1, PEn2, PEn3, PFn1, PFn2, and PFn3 by combining nine oligomers. As shown in Figure 2, the unit_A_ conjugated area extended of the oligomers for each line from left to right, and the unit_D_ conjugated area extended of the oligomers for each column from top to bottom. The nine molecules are evidently periodic variational in structure, and PDn2, PEn1, PEn2, PEn3, and PFn2 are asymmetric structures. In what follows, we will discuss the relationship between the structure and properties of these ideal molecules. 

### 3.2. Geometric Optimization and Electronic Structure

**Figure 3:**
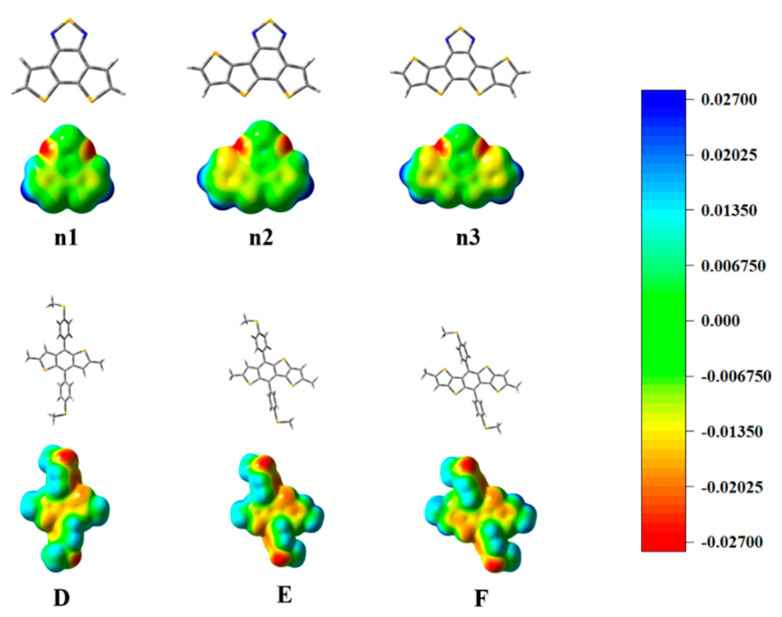
The optimized geometry and ESP distributions (electron density isosurface, 0.001 au) on the n1, n2, n3, D, E, and F models.

The optimized geometry at the ground state of the unit_D_ and unit_A_ moieties exhibited good planarity, as shown in Supplementary Materials Figure S1. The calculated frontier molecular orbits (FMOs) of the highest occupied molecular orbital (HOMO), the lowest unoccupied molecular orbital (LUMO) energy levels are shown in Supplementary Materials Figure S2. To deeply understand the charge distribution of the structures of the unit_D_ and unit_A_ moieties, the ESP was calculated and plotted, as shown in Figure 3. The blue and red color represent the high and low potential, respectively, which means the absence and abundance of electrons in this area, respectively. Intermediary transition colors depict the neutral electrostatic potentials. The results indicate that the conjugated thiophenes regions are highly electron-rich. Most of its positive regions are mainly localized at the hydrogen atoms in the non-conjugated methyl groups. The location of the electron-rich sulfur atoms is also evident, and they appear yellowish around these regions. For the moieties n1, n2, and n3, the N atoms connected directly to the S atoms, which attract considerable electron density. The electronegativity difference of atoms led to a redistribution of charge. 

**Figure 4:**
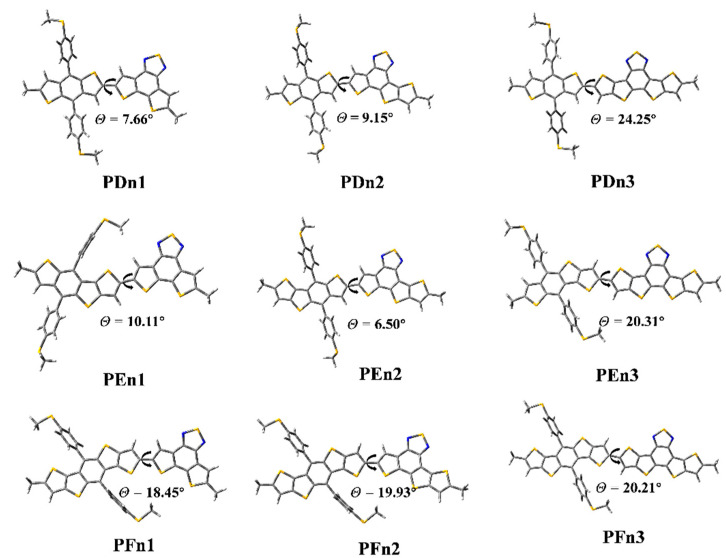
The optimized geometry of the oligomers.

As shown in Figure 4, the ground state geometry of oligomers was optimized. The dihedral angles (*θ*) between the two moieties of PDn1, PDn2, and PDn3 are 7.66°, 9.15°, and 24.25°, respectively. The *θ* of PFn1, PFn2, PFn3 are 18.45°, 19.93°, and 20.21°, respectively. The *θ* increased with the π-extended among the PDn and PFn series oligomers; it is very likely that the expansion of the conjugate area leads to the enhanced rigidity. This phenomenon also appeared among PEn1 and PEn3, as 10.11° and 20.31° respectively. The size of the conjugation region also significantly affected the molecular planarity. However, the *θ* (PEn2) was only 6.50°; it was compounded by two asymmetric units (E and n2). The PEn2 with the minimum dihedral angles will be beneficial for intermolecular π–π stacking, which may produce good molecular stacking morphology among the BHJ PSCs [55].

**Figure 5:**
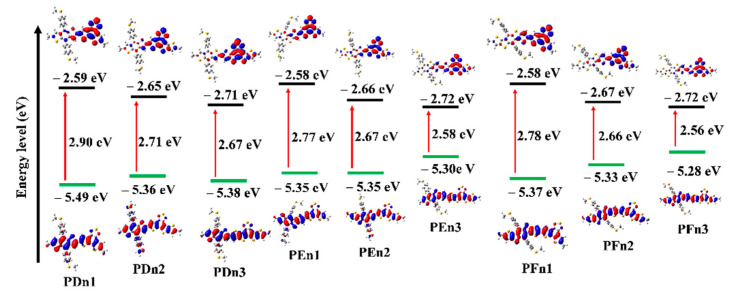
Calculated frontier molecular orbital for all the molecules.

The values of HOMO and LUMO energy levels, and the energy difference (ΔH-L) between HOMO and LUMO values of the studied oligomers in solvent phases were also calculated and are presented in Table 1 and Figure 5. The HOMO increased, but LUMO declined with the moieties in the π–π conjugation region that extended gradually, which resulted in the decreased ΔH-L values. It means the π-extended strategy could effectively lower the LUMO and improve the HOMO simultaneously. The π-extended strategy could be applied to regulate the molecular energy level. Figure 5 distinctly shows the electron cloud density transition from HOMO to LUMO, which could be attributed to the ICT from donor unit to acceptor unit and the π–π* transition of the oligomers. It also indicates their good charge transfer capacity to a certain extent. 

**Figure 6:**
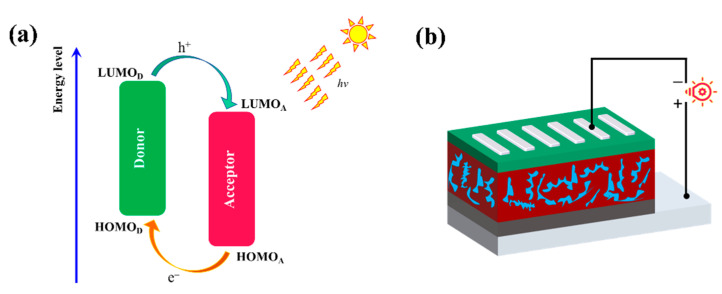
(**a**) Schematic illustration of the energy levels at the interface of polymer/ITIC pair in PSCs. (**b**) Device structure.

The PCE of the PSCs can be calculated by Equation (1) [56]:

(1) $$\mathrm{PCE}(\%)=\frac{J_{SC}\times V_{OC}\times FF}{P_{in}}\times 100\%$$

where *J*_SC_, *V*_OC_, *FF*, and *P_in_* are short-circuited current density, open-circuit voltage, fill factor, and incident light intensity, respectively. The illustration of the working mechanism of the D/A blend and the device structure are displayed in Figure 6. The voltage is proportional to PCE and a higher *V*_OC_ would be of benefit to achieve a high PCE, which could be estimated by Equation (2) [56]:

(2) $$V_{OC}=\frac{1}{e}(E_{\mathrm{HOMO}}^{\mathrm{Donor}}-E_{\mathrm{LUMO}}^{\mathrm{Acceptor}})-0.5V$$

where $E_{\mathrm{HOMO}}^{D}$ and $E_{\mathrm{LUMO}}^{A}$ represent the energy level of HOMO (donor) and LUMO (acceptor), respectively. The value of 0.5 V is the non-fullerene PSCs empirical factor of efficient charge separation [57]. In this paper, a well-known NAFs ITIC was selected [57] and calculated under the B3LYP-D3(BJ)/6-311G(d) level. The calculated energy levels of ITIC are −5.64 eV (HOMO) and −3.56 eV (LUMO). The ITIC optimized geometry, energy level, and orbital electron cloud density distribution are shown in Supplementary Materials Figure S3. The calculated *V*_OC_ are summarized in Table 1. 

### 3.3. Spectral Properties

**Figure 7:**
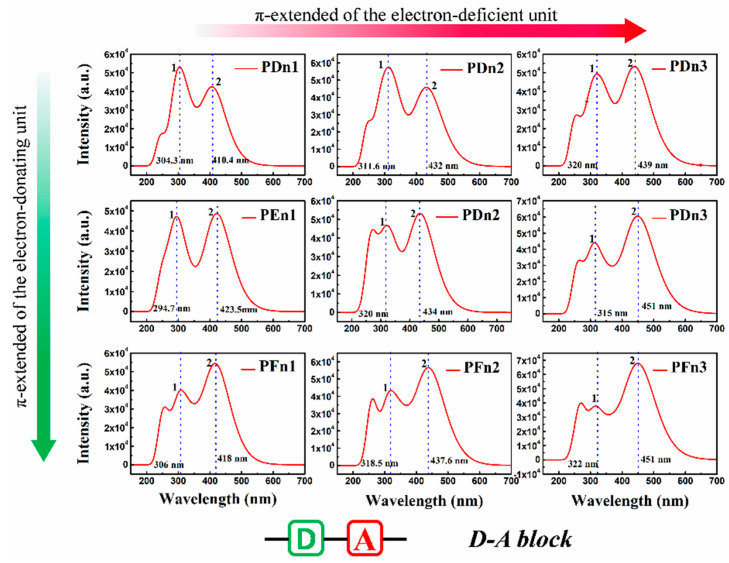
The simulated absorption spectrum for PDn1, PDn2, PDn3, PEn1, PEn2, PEn3, PFn1, PFn2, and PFn3. The value of the FWHM is 0.333 eV.

For organic semiconductor materials in PSCs, the sunlight absorbance and absorption spectrum of molecules play a vital role in the photoelectric conversion process. To deeply investigate the optical properties of these molecules, the light absorption spectra were simulated, as shown in Figure 7. All the nine spectra also expend two main absorption areas located at ~300 nm (peak 1) and 400~460 nm (peak 2). As an instance of PDn1, PDn2, and PDn3, peak 2 redshifts move from 410.4 nm to 439 nm, but peak 1 shifts slightly. Compared with PDn1, PDn2, and PDn3, the peak 2 redshift is mainly attributed to the π-extended unit_A_. According to molecular orbital theory, the π–π conjugation extended region could increase the energy of π orbital, but lower the π* and lower the energy gap (E_g_), which correspond well to Table 1. The higher light-harvesting efficiency (LHE) value of the molecules would be of benefit for the high *J*_SC_ of the PSCs devices. The corresponding LHE can be calculated by Equation (3) [58]:

(3) $$\mathrm{LHE}=1-10^{-f}$$

where *f* is the oscillator strength of the donor molecule. The higher *f* of the transition suggests a larger absorption coefficient [59]. The results of the calculations indicate that, with the increase in the conjugation region, the LHE increased, and PFn3 has the maximum LHE 0.975. Other crucial spectroscopic parameters, such as the vertical excitation energies and compositions of vertical transitions in terms of molecular orbital, were carried out through TD–DFT calculations. The corresponding results were extracted and are summarized in Table 2. 

According to the PSCs photoelectric conversion mechanism, the excitons were generated by donor materials after absorbing light, then diffused to the D/A interface and, lastly, split into electrons and holes. Nevertheless, the excitons were bounded by the Coulomb attraction and would not directly split into free charges. Generally, the Coulomb attraction is defined as exciton binding energy (*E*_b_). The *E*_b_ could be estimated by Equation (4) [60]:

(4) $$E_{b}=E_{g}-E_{s1}$$

In addition, the amount of excitons that arrive at the D/A interface is determined by the exciton diffusion length (*L*_D_). Generally, a long exciton lifetime *τ* (ns) corresponds to a long *L*_D_ of the material in a solid film. In this paper, *τ* could be evaluated by the Einstein spontaneous emission relationship [61]:

(5) $$\tau =1.499\times \frac{1}{f\times E_{i}{{}_{f}}^{2}}$$

where in *E_if_* is the excitation energy in cm^−1^. Based on Equation (5), a lower *f* and *E_if_* will lead to a larger *τ*. The calculated *τ* results are summarized in Table 2; PDn2 had the longest *τ* = 2.56 ns. This means that the excitons produced by PDn2 have a higher probability of arriving at the D/A interface under the circumstance of neglecting other factors, which would be of benefit to achieve a high PCE.

### 3.4. Ionization Potential (IP) and Electron Affinity (EA)

The holes/electrons transport barriers of the active layer in the PSCs can be determined by the vertical ionization potentials (IP) and electron affinities (EA) of the molecule, which is an important reference for molecular design. Ordinarily, a small IP could lower the hole injection barrier, and a larger EA makes the electron injection easier [62]. The IP and EA of the molecules could be calculated by Equations (6) and (7):

(6) $$IP=E_{+}-E_{0}$$

(7) $$\mathrm{EA}=E_{0}-E_{-}$$

The calculated *IP/EA* valances result of the nine molecules indicated close relations with their unit_D_/unit_A_ conjugate area sizes. For instance, the *IP* valances of PDn1, PEn1, and PFn1 are 5.56 eV, 5.42 eV, and 5.44 eV, respectively, while the EA valances are almost unchanged with the π-extended of the unit_D_. This indicates that the IP valances are in intimate connection with the unit_D_. PEn1 is an asymmetric molecule for their block with asymmetry unit_D_. Meanwhile, compared with PDn1, PDn2, and PDn3, the EA valances increased with the π-extended of the unit_A_. The results suggested the IP and EA values could be regulated by changing the magnitude of the conjugate region. It could be a useful way to design PSCs donor materials.

### 3.5. Reorganization Energy

The excitons generated by the donor materials diffused and arrived at D/A interface, which split into electrons and holes under the energy difference of the donor and acceptor. Then, the electron/hole was transmitted in the double continuous interpenetrating network active layer and collected by the corresponding electrode in the terminal. The charge transfer mechanism of the active layer is an incoherent hopping model, which could be described as electron/hole transport only between adjacent molecules. The charge transfer rate constants (*k*) can be calculated by Equation (8) [62], based on the semiclassical Marcus theory:

(8) $$k=V^{2}\sqrt{\frac{\pi }{h^{2}k_{B}T\lambda }}\exp(-\frac{\lambda }{4k_{B}T})$$

**Figure 8:**
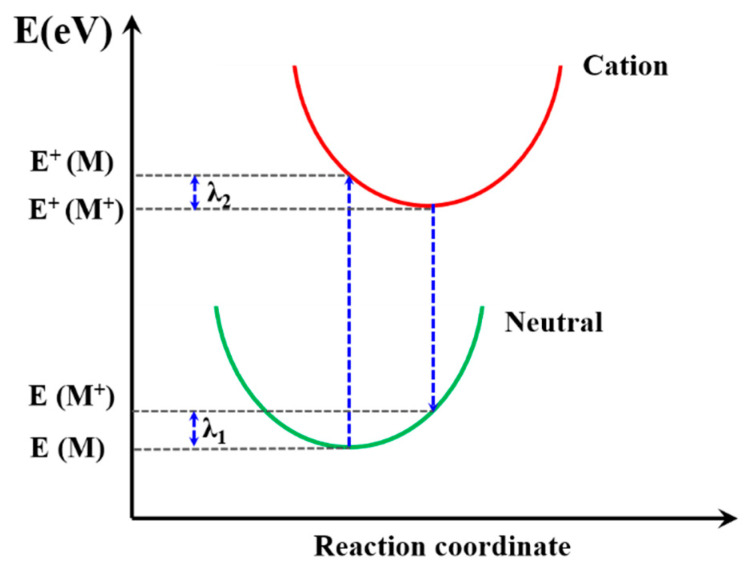
Schematic plot of reorganization energy.

The reorganization energy (λ) is closely connected with the mobilities of organic materials [63] so that the λ is significant to predict the charge extract property within PSC device. It is the sum of two energy terms, as shown in Figure 8, and λ_1_ and λ_2_ could be described as [62]:

(9) $$\lambda_{1}{=E\;(M}^{+})-E\;(M)$$

(10) $$\lambda_{2}{=E}^{+}\left(M\right)-E^{+}{(M}^{+})$$

where E(M^+^) and E(M) are the energy of neutral molecules at the cationic structure and neutral ground state, respectively. The E^+^(M) and E^+^(M^+^) are the energy of the cation at the optimized structure of the neutral molecule and cation structure, respectively. The calculated λ results are summarized in Table 3; PFn1 has the maximum λ 0.531 eV, and PDn2 and PEn2 have the near minimum λ, 0.180 eV and 0.181 eV, respectively. According to Equation (8), the lower λ is, the higher *k* will be. It can be seen that, except PFn2, all the asymmetric molecules have a comparatively low λ [64,65,66,67]. This means that these asymmetric molecular skeletons in charge carrier transport have advantages in comparison with symmetry skeletons.

## 4. Conclusions

In this work, three new electron-deficient units (n1, n2, and n3) and three electron-donating units (D, E, and F) were elaborately designed, and nine oligomers were obtained by combining different π-conjugation area unit_A_ and unit_D_. The structure–performance relationships of the nine oligomers were systematically investigated by the quantum mechanical calculations. The nine symmetry or asymmetry molecule structures have been calculated at the B3LYP-D3(BJ)/6-311G(d) level and using chloroform as the solvent. The results of the DFT calculations indicate that asymmetry molecule PEn2 has the minimum dihedral angle among all the molecules, which points to the advantages in the molecular stacking of asymmetric structures. The energy level and E_g_ also periodically changed along with the molecule structure periodic variation. The results of the TD–DFT calculations indicate that the spectral absorption region/peak could be regularly affected by changing the π-extended size. The size of the conjugate region of the unit_D_/unit_A_ has close relation with IP/EA values. The calculated reorganization energy also demonstrated that asymmetric molecules PDn2 and PEn2 are more conducive to realize a higher PCE, because of their lower λ. The results certified that the asymmetric structure could be of benefit to the charge extraction within the PSCs device. There are still some limitations to accurately predict the real material properties in the application of the complex in real bulk-heterojunction solar cells, but our work has illustrated the advantages of asymmetric molecules, which also can be applied to molecule design. 

## Figures and Tables

**Table 1:** The optimized geometry of oligomers: dihedral angles (*θ*) of the D-A block, energies of HOMO, LUMO, *E*_g_ values, and *V*_OC_.

Molecule	*θ* (°)	HOMO (eV)	LUMO (eV)	*E*_g_ (eV)	*V*_OC_ (V)
PDn1	7.66	−5.49	−2.59	2.90	1.35
PDn2	9.15	−5.36	−2.65	2.71	1.22
PDn3	24.25	−5.38	−2.71	2.67	1.24
PEn1	10.11	−5.35	−2.58	2.77	1.21
PEn2	6.50	−5.33	−2.66	2.67	1.19
PEn3	20.31	−5.30	−2.72	2.58	1.16
PFn1	18.45	−5.37	−2.58	2.78	1.23
PFn2	19.93	−5.33	−2.67	2.66	1.19
PFn3	20.21	−5.28	−2.72	2.56	1.14

**Table 2:** The table shows the absorption wavelength (λ_max_), vertical transition energies (eV), main transition contribution, the oscillator strengths (*f*) of all the molecules, the lifetime of spontaneous radiation *τ*(ns), and light-harvesting efficiencies (LHE).

Molecule	State	Composition ^a^	E (eV)	λ_abs_ (nm)	E_b_ (eV)	*f*	*τ* (ns)	LHE
PDn1	S0→S1	H→L (78.3%)	3.02	410.40	0.12	1.0063	2.51	0.901
	S0→S2	H→L + 1 (55.6%)	3.66	339.06				
PDn2	S0→S1	H→L (75.0%)	2.85	434.39	0.14	1.1058	2.56	0.922
	S0→S2	H→L + 1 (47.0%)	3.55	349.30				
PDn3	S0→S1	H→L (79.7%)	2.80	442.02	0.13	1.2878	2.27	0.948
	S0→S2	H→L + 1(61.7%)	3.52	352.15				
PEn1	S0→S1	H→L (77.0%)	2.93	423.32	0.16	1.1767	2.28	0.933
	S0→S2	H→L + 1 (71.3%)	3.55	348.97				
PEn2	S0→S1	H→L (76.7%)	2.83	438.25	0.16	1.2817	2.24	0.947
	S0→S2	H→L + 1 (55.2%)	3.49	354.74				
PEn3	S0→S1	H→L (79.6%)	2.74	452.55	0.16	1.4560	2.10	0.965
	S0→S2	H→L + 1 (66.8%)	3.38	366.57				
PFn1	S0→S1	H→L (76.4%)	2.95	420.78	0.17	1.2804	2.07	0.947
	S0→S2	H→L + 1 (67.9%)	3.53	350.71				
PFn2	S0→S1	H→L (79.4%)	2.81	441.75	0.15	1.3501	2.16	0.955
	S0→S2	H→L + 1 (66.3%)	3.45	358.92				
PFn3	S0→S1	H→L (76.7%)	2.73	454.02	0.17	1.6127	1.92	0.975
	S0→S2	H→L + 1 (65.8%)	3.33	372.74				

^a^ H represents HOMO; L represents LUMO.

**Table 3:** Calculations of vertical ionization potentials (IP), electron affinities (EA), and reorganization energy (λ).

**Molecule**	**IP (eV)**	**EA (eV)**	**λ1 (eV)**	**λ2 (eV)**	**λ (eV)**
PDn1	5.56	2.34	0.086	0.393	0.479
PDn2	5.40	2.40	0.068	0.112	0.180
PDn3	5.44	2.46	0.064	0.378	0.442
PEn1	5.42	2.33	0.058	0.291	0.349
PEn2	5.38	2.42	0.070	0.111	0.181
PEn3	5.37	2.48	0.084	0.350	0.434
PFn1	5.44	2.35	0.125	0.406	0.531
PFn2	5.39	2.43	0.126	0.392	0.518
PFn3	5.34	2.49	0.094	0.350	0.443

## Data Availability

All datasets generated for this study are included in the article.

## Supplementary Materials

The following are available online at https://www.mdpi.com/article/10.3390/ma14216723/s1: Figure S1: The side view of the optimized geometry of the monomers at ground state; Figure S2: The HOMO and LUMO orbital electron cloud distribution of the monomers; Figure S3: The optimized geometry at ground state and HOMO and LUMO orbital electron cloud distribution of the ITIC.
