# Supplementary material for: Rational Design and Characterization of Symmetry-Breaking Organic Semiconductors in Polymer Solar Cells: A Theory Insight of the Asymmetric Advantage
Source: Materials (Basel). 2021 Nov 8;14(21):6723. doi: 10.3390/ma14216723 (PMC8587437; doi:10.3390/ma14216723)
Supplement: Supplementary file 1 [file materials-14-06723-s001.zip › materials-1439148-supplementary.pdf]

Article

Rational Design and Characterization of Symmetry-Breaking Organic Semiconductors in Polymer Solar Cells: A Theory Insight of the Asymmetric Advantage

Zezhou Liang [1], Lihe Yan [1,*], Jinhai Si [1], Pingping Gong [1,2], Xiaoming Li [3], Deyu Liu [4], Jianfeng Li [2] and Xun Hou [1]

1 Key Laboratory of Physical Electronics and Devices of the Ministry of Education & Shaanxi Key Lab of Photonic Technique for Information, School of Electronic Science and Engineering, Faculty of Electronic and Information Engineering, Xi'an Jiaotong University, Xi'an 710049, China; zezhouliang@foxmail.com (Z.L.); jinhaisi@mail.xjtu.edu.cn (J.S.); gpbxnl@163.com (P.G.); houxun@mail.xjtu.edu.cn (X.H.)
2 School of Materials Science and Engineering, Lanzhou Jiaotong University, Lanzhou 730070, China; ljfpyc@163.com
3 School of Chemistry, Beihang University, Beijing 100191, China; lixiaoming617@hotmail.com
4 Department of Materials Science and Engineering, Ocean University of China, Qingdao 266100, China; lunlunyu@126.com
* Correspondence: liheyan@mail.xjtu.edu.cn; Tel.: +86-029-82665670

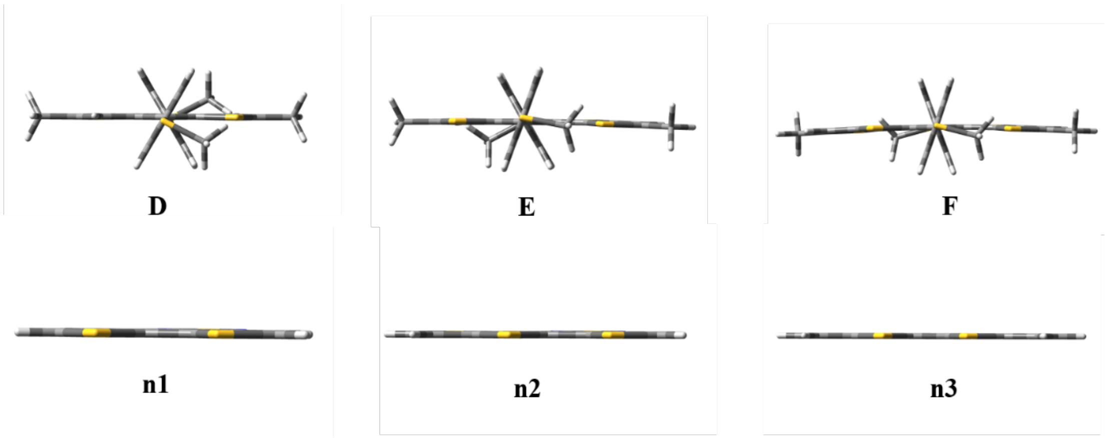

Figure S1. The side view of the monomers optimized geometry at ground state.

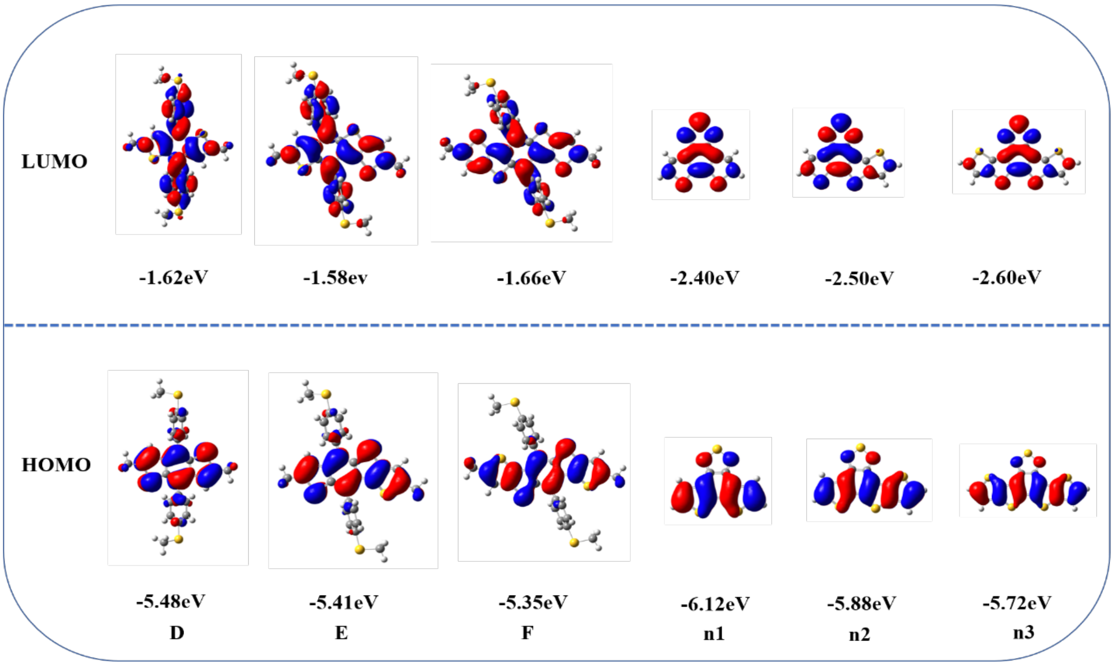

Figure S2. The HOMO and LUMO orbital electron cloud distribution of the monomers.

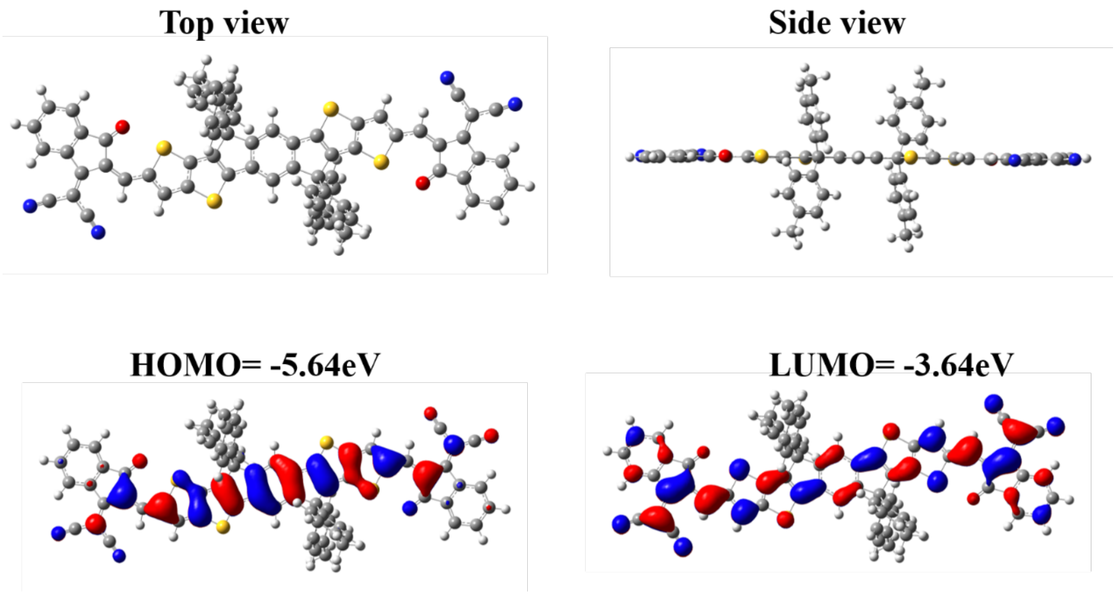

Figure S3. The optimized geometry at ground state and HOMO and LUMO orbital electron cloud distribution of the ITIC.
